# *Weizmannia coagulans* JA845 Postbiotics Alleviate Atherosclerosis via TMAO-Related Gut Microbiota Regulation and JAK/STAT3 Pathway Inhibition

**DOI:** 10.3390/nu17193027

**Published:** 2025-09-23

**Authors:** Liying Ma, Nan Li, Zijian Zhao, Yujuan Zhao, Ge Yang, Lei Zhao, Shengyu Li

**Affiliations:** 1 Institute of Agro-Food Technology, Jilin Academy of Agriculture Sciences (Northeast Agriculture Research Center of China), Changchun 130033, China; liyingma1130@163.com (L.M.);; 2 School of Food Science and Technology, Jiangnan University, Wuxi 214122, China; 3 School of Pharmaceutical Sciences, Changchun University of Chinese Medicine, Changchun 130117, China

**Keywords:** postbiotics, *Weizmannia coagulans* JA845, atherosclerosis, gut microbiota, TMAO metabolism

## Abstract

**Objectives:** Postbiotics have been shown to significantly attenuate atherosclerosis development. This study aimed to elucidate the mechanisms underlying this protective effect, focusing on gut microbiota remodeling, reduction of trimethylamine *N*-oxide (TMAO), and suppression of the TMAO-activated inflammatory pathway. **Methods:** A high-fat diet (HFD) combined with choline was used to establish an atherosclerosis mouse model. Mice were divided into four groups: control, model, JA845, and Post-JA845 groups. Histological analysis, immunofluorescence staining, inflammatory cytokine detection, 16S rRNA sequencing, metabolomics, and proteomics were used to evaluate the regulatory effects of JA845 postbiotics on gut microbiota composition, TMAO metabolism, and the JAK/STAT3 signaling pathway. **Results:** Histopathological examination revealed that JA845 postbiotics markedly attenuated atherosclerotic plaque formation in the aorta and improved overall vascular pathology. The treatment effectively regulated lipid metabolism, demonstrating significant reductions in atherogenic LDL and total cholesterol levels, while promoting beneficial HDL elevation. JA845 postbiotics demonstrated potent anti-inflammatory effects by significantly lowering circulating levels of IL-6, IL-33, IL-1β, and TNF-α. Gut microbiota analysis showed substantial compositional changes, with increased abundance of beneficial *Bacteroides* and *Parabacteroides* alongside decreased pro-atherogenic *Ruminococcus* and *Akkermansia*. At the molecular level, the postbiotics inhibited TMAO generation, suppressed JAK/STAT3 signaling pathway activation, and enhanced endothelial function through upregulated eNOS-mediated nitric oxide production. These coordinated effects collectively contribute to the observed cardiovascular protection. **Conclusions:** JA845 postbiotics exhibit superior efficacy in reducing TMAO levels, modulating gut microbiota, alleviating inflammation, and improving vascular function, offering a novel strategy for atherosclerosis prevention and treatment.

## 1. Introduction

Postbiotics are emerging as a novel and promising alternative to probiotics. They are derived from the inactivation and decomposition of probiotics [1]. In 2021, the term “postbiotics” was formally defined to include inanimate commensal bacteria, cell-free supernatants, and other key components that contribute to host health [2]. Postbiotics consist of a diverse array of components, including inactivated microbial cells, cell wall fragments, peptides, short-chain fatty acids (SCFAs), and other bioactive compounds [3]. These components have been shown to offer several health benefits. They can modulate the gut microbiota, helping to maintain a balanced and healthy microbial community. Postbiotics can also regulate immune responses, contributing to a well-functioning immune system. Additionally, they exert anti-inflammatory and antioxidant effects, which are crucial for overall health [4,5]. Postbiotics exert metabolic regulation through coordinated mechanisms, initiating with hepatic GPR41/43 activation that simultaneously suppresses lipogenic enzymes while enhancing PPARα-dependent fatty acid β-oxidation [6], ultimately normalizing serum lipid profiles [7]. Furthermore, postbiotics attenuate plaque formation by modulating the gut microbiota-TMAO axis to suppress foam cell generation while activating the LXRα-ABCA1 pathway to enhance cholesterol efflux [8]. Due to their non-living nature [9], postbiotics eliminate concerns regarding colonization ability, antibiotic resistance transfer, and potential infections, making them a promising alternative to probiotics in functional foods and therapeutics [10,11].

Trimethylamine *N*-oxide (TMAO) is produced when dietary choline and L-carnitine are metabolized by specific gut microbiota (including *Anaerococcus*, *Clostridium*, and *Desulfovibrio*) into trimethylamine (TMA), which is subsequently oxidized in the liver by flavin-containing monooxygenases (FMOs) [12]. The production of TMA varies significantly depending on gut microbiota composition and the activity of choline TMA-lyase, consequently affecting TMAO levels [13]. Research has demonstrated that elevated TMAO levels are closely associated with inflammation [14,15], endothelial dysfunction, and the development of CVDs [16]. Recent studies indicate that postbiotics may regulate the TMAO metabolism through the following mechanisms: (1) competitively inhibiting choline TMA-lyase activity in TMA-producing bacteria, (2) remodeling gut microbiota to promote the growth of beneficial species (e.g., Bifidobacterium and Lactobacillus) [17,18], or (3) enhancing intestinal barrier function to reduce TMA absorption [14]. Furthermore, TMAO exacerbates metabolic disorders, including obesity, dyslipidemia, and diabetes, by promoting insulin resistance (via NF-κB-mediated inflammation), disrupting bile acid metabolism, and impairing gut barrier integrity, ultimately leading to systemic low-grade inflammation [19]. Therefore, targeting the gut microbiota-TMAO axis through postbiotic intervention represents a promising therapeutic strategy for mitigating atherosclerosis and metabolic diseases, although further mechanistic investigations are required.

*Weizmannia coagulans* are spore-forming, lactic acid-producing bacteria known for their antioxidant [20], anti-inflammatory, anti-angiogenic, and anti-cancer properties [21], while probiotics have been shown to influence lipid metabolism and cardiovascular health [22]. However, the extent to which *W. coagulans*-derived postbiotics can exert similar or even superior effects through microbial-derived metabolites and immune regulation remains an open question. Our previous research has demonstrated that *W. coagulans* JA845 possesses a wide range of bioactivities, including potent anti-inflammatory effects, immunomodulatory capacity, antioxidant potential, and the ability to regulate lipid metabolism [16,19,20]. Despite these promising findings, the impact of *W. coagulans* JA845 postbiotics on gut microbiota has not been thoroughly investigated. Therefore, the study analyzes the efficacy of *W. coagulans* probiotics and their derived postbiotics, evaluating the advantages of postbiotics in terms of stability and safety, as well as their lipid-regulating and immunomodulatory effects. Meanwhile, by combining gut microbiota and metabolite analysis, it elucidates the remodeling effect of JA845-derived postbiotics on microbiota structure and their association with beneficial metabolites. By conducting a detailed analysis of vascular inflammatory injury pathways and targeted profiling of gut microbial metabolism, we aim to elucidate the mechanisms underlying the modulation of atherosclerosis by *W. coagulans* JA845 and its probiotic formulation via the gut-vascular axis. This research is anticipated to enhance our comprehension of postbiotic mechanisms and bolster the development of postbiotics as safe and effective functional ingredients for disease prevention and treatment.

## 2. Materials and Methods

### 2.1. W. coagulans JA845 Culture and Postbiotic Preparation

The *W. coagulans* JA845 strain originates from the China General Microbiological Culture Collection Center (CGMCC, Changchun, China) with the preservation number 19576. The strain is cultured in Luria–Bertani (LB) medium containing specific nutrients (tryptone 10 g/L, yeast extract 5 g/L, sodium chloride 10 g/L, and glucose 10 g/L) and incubated at 50 °C, 180 rpm for 24 h. Next, the *W. coagulans* JA845 precipitate was resuspended in physiological saline and adjusted to a dose of 1.0 × 10^9^ colony-forming units (CFU) per mouse per day for intragastric administration.

The *W. coagulans* JA845 suspension (1.0 × 10^9^ CFU/mL) was subjected to ultrasonic disruption (800 W, 15 min) in an ice bath. The resulting lysate was adjusted back to the original bacterial concentration using sterile distilled water for direct use in animal studies.

### 2.2. Animal Experiments

**Figure 1:**
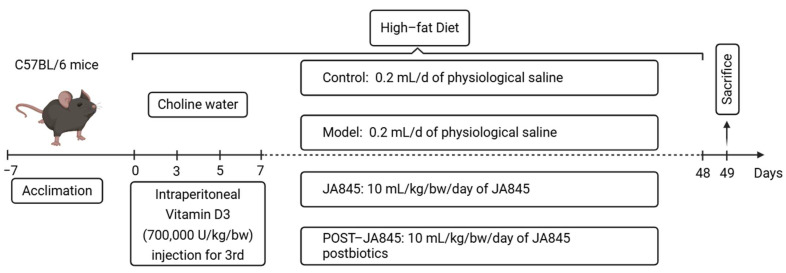
Experimental procedure and treatment schedule (Created in BioRender. Ma, L. (2025) https://BioRender.com/1w4q5yh).

A total of 40 female C57BL/6 mice (18–20 g, 8-week-old) were obtained from Yisi Laboratory Animal Technology (Changchun, China). The animal certification number is SCXK (Liao) 2020-0001. Mice were acclimated for one week on a standardized diet to ensure metabolic stability. They were housed in a controlled setting maintained at 22 ± 1 °C, with alternating 12-h periods of light and darkness. All experimental protocols adhered to approval from the Research Ethics Board at the Jilin Academy of Agricultural Sciences (Northeast Agricultural Research Center of China) (No. 202501, Approval Date: 1 January 2023, Changchun, China). The mice were randomly divided into four groups (n = 10). The control group received a regular diet and intraperitoneal injection of 10 mL/kg/bw/d of PBS buffer. The model group, the JA845 group, and the Post-JA845 group were continuously fed a high-fat diet (HFD) and received intraperitoneal injection of VD3 (700,000 U/kg/bw) on the 3rd, 5th, and 7th days while drinking customized water containing 1.0% choline. The control group and the model group were administered with 0.2 mL/d of physiological saline by gavage; the JA845 group was gavaged with *W. coagulans* JA845 at a concentration of 1.0 × 10^9^ CFU/mL at a dosage of 10 mL/kg/bw/day; and the Post-JA845 group was administered with 10 mL/kg/bw/day of *W. coagulans* JA845 postbiotics (equivalent to containing 1.0 × 10^9^ CFU/mL of *W. coagulans* JA845). The mice underwent continuous treatment for 6 weeks. After the final dose was administered, they were fasted for 12 h and blood was collected. Serum, aorta, and cecal feces were processed and stored at −80 °C (Figure 1).

### 2.3. Histological Examination

Hematoxylin and eosin (H & E) were used to stain the altered pathological features of the abdominal aorta of atherosclerotic mice. The abdominal aorta of each mouse was removed, and the samples were fixed in 10% paraformaldehyde fixative for 24 h, frozen, and subsequently embedded in paraffin for 48 h. The processed samples were then cut into 5 μm thin sections using a microtome. The tissue sections were stained with H & E. The plaque area at the root of the abdominal aorta was observed under a 200× optical microscope.

### 2.4. Immunofluorescence

The fixed abdominal aortic tissues were embedded in paraffin, frozen at −20 °C, and then sliced. The slices were first incubated with a solution of PBS (pH = 7.4) containing 3% BSA and 0.3% Triton X-100. Subsequently, they were co-incubated with CD68 antibody (Servicebio, Wuhan, China, GB113109, 1:400) and α-SMA antibody (Servicebio, GB111364, 1:500) at 4 °C for 12 h. After that, the slices were incubated with the secondary antibody (Servicebio, GB21301, 1:300) at room temperature for 1 h. Finally, they were incubated with diaminobenzidine for approximately 10 min. The digital pathological images were analyzed using Aipathwell (V2) analysis software, which automatically calculated the results for various items based on the original data and algorithm formulas. The positive cell rates for transmembrane glycoprotein (CD68) and α-smooth muscle actin (α-SMA) were calculated (red light positive rate = total number of red-light positive cells/total number of cells; green light positive rate = total number of green light positive cells/total number of cells).

### 2.5. Biochemistry Analysis

The mouse plasma was allowed to stand at 4 °C for 1 h and then centrifuged (4000 rpm, 4 °C, 10 min), and the supernatant was collected and frozen at −80 °C for use. The levels of TG, TC, HDL, LDL, TNF-α, IL-6, IL-1β, and IL-33 in serum were detected by using the commercial enzyme-linked immunosorbent assay (ELISA) (Jiangsu Meibiao Biotechnology Co. Ltd., Yancheng, China). Additionally, the levels of BAs in the serum were detected using kits (Nanjing Jiancheng Bioengineering Institute, Nanjing, China).

### 2.6. Western Blotting

Protein immunoblotting was used to detect the protein expression levels of FMO3 in liver tissue and JAK and STAT3 in abdominal aorta. Total proteins were extracted by adding lysis buffer (RIPA:PMSF:protease inhibitor = 100:1:1), and the protein content was determined by the BCA method. After electrophoresis and membrane transfer, the proteins were blocked with 5% BSA at room temperature for 1 h. The primary antibody was incubated overnight at 4 °C, and the secondary antibody was incubated in the dark at 37 °C for 1 h. Luminescence was developed using an Image Quant LAS 4000 (Clinx, Shanghai, China) machine to standardize the protein content, and the grey values of the target bands were calculated using Image J (1.50i) software.

### 2.7. High-Throughput Sequencing of 16S rRNA in Cecum Contents

After collecting fecal samples from mice, we employed the QIAamp Fast DNA Fecal Mini Kit from QIAGEN to isolate and purify total DNA. Specific primers targeting the V3-V4 regions of bacterial 16S rRNA were designed, with sequences of F: ACTCCTACGGGAGGCAGCA and R: GGACTACHVGGTWTCTAAT, and PCR amplification was conducted. Subsequently, the PCR products were sequenced on the Illumina MiSeq platform, and sequences with a homology of over 97% were categorized into operational taxonomic units (OTUs). Finally, we conducted a thorough analysis of the diversity of the intestinal microbiota using the QIIME tool (version 1.9).

### 2.8. Analysis of Differential Metabolites

#### 2.8.1. Metabolite Extraction

The fecal sample was taken out from −80 °C; 100 mg of the sample was weighed after liquid nitrogen grinding, and 10 μL of internal standard and 500 μL of pre-cooled acetonitrile/water solution (9/1, *v*/*v*) were added. Black ceramic beads were added, homogenized twice (20 s each), vortexed for 30 s, and incubated at 4 °C for 10 min to precipitate the protein. They were then centrifuged at 10,000 rpm, 4 °C, 20 min. The supernatant was collected and freeze-dried, and the samples were stored at −80 °C.

#### 2.8.2. LC-MS Detection Conditions

TMA, TMAO, Betaine, Creatinine, Carnitine, and Choline in the samples were quantitatively analyzed using an Ultra-high Performance Liquid Chromatography-Mass Spectrometry (UHPLC-MS) system. The samples were effectively separated by the Agilent 1290 Infinity system at 25 °C. The mobile phase was composed of an aqueous solution (A) composed of 10 mM ammonium format and 0.4% formic acid and an acetonitrile solution (B) with 0.4% formic acid; the flow rate was maintained at 400 μL/min, and the sample volume was 2 μL for each injection. To achieve baseline separation of TMA and TMAO, a gradient elution program was used: 0–1.5 min, 95% (B); 1.5–7.0 min, 95–85% (B); 7.0–7.1 min, 85–50% (B); 7.1–10 min, 50% (B); 10–10.5 min, 50–90% (B); and 10.5–14.5 min, 90% (B). The mass spectrometry detection was carried out using 5500 QTRAP mass spectrometer (AB SCIEX, Framingham, USA) in positive ion mode. The conditions of the electrospray ionization (ESI) were as follows: source temperature: 550 °C; Ion Source Gas1 (Gas1): 55; Ion Source Gas2 (Gas2): 55; Curtain gas (CUR): 40; and ion sapary voltage floating (ISVF): +4500 V. Multi-Reaction Monitoring (MRM) mode was used to detect the target ion pairs, ensuring the accuracy and sensitivity of the analysis.

### 2.9. Statistical Analysis

The data were analyzed using GraphPad Prism 8 software. Statistical results were expressed as means ± S.D. One-way analysis of variance (ANOVA) with Tukey’s multiple comparisons was employed for statistical analysis, with a significance level of *p* < 0.05 considered as statistically significant.

## 3. Results

### 3.1. W. coagulans JA845 Postbiotics Regulates the Serum Lipid Levels in AS Mice

**Figure 2:**
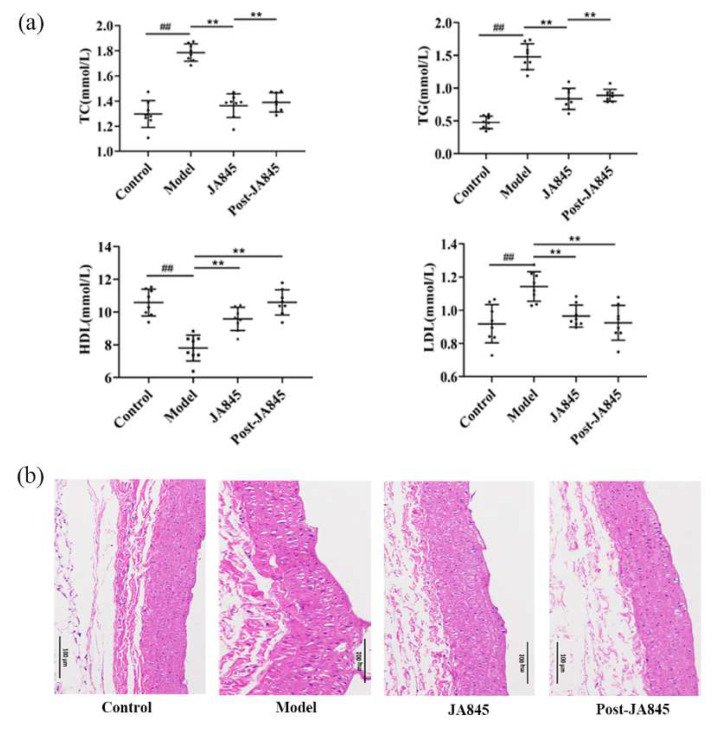
*W. coagulans* JA845 postbiotics protect the abdominal aorta from injury by regulating lipid levels (n = 8). (**a**) TG, TC, HDL, and LDL were measured after *W. coagulans* JA845 postbiotics treatment. (**b**) Effects of *W. coagulans* JA845 postbiotics administration on attenuated histopathological features of the aorta in mice (200×). Results are manifested as the mean ± SD. Compared with the control group, ## *p* < 0.01, mean ± SD; compared with the model group, ** *p* < 0.01, mean ± SD.

The effects of *W. coagulans* JA845 postbiotics on serum lipid levels in mice with AS are illustrated in Figure 2a. Following a 6-week intervention period, the model group exhibited a significant decrease in serum high-density lipoprotein (HDL) levels, whereas the levels of low-density lipoprotein (LDL), triglycerides (TG), and total cholesterol (TC) were significantly elevated (*p* < 0.01). In contrast, after supplementation with *W. coagulans* JA845 postbiotics, the HDL level was significantly increased compared to the model group (*p* < 0.01), and concurrently, the levels of LDL, TG, and TC were significantly reduced in the treatment group (*p* < 0.01).

### 3.2. W. coagulans JA845 Postbiotics Improves the Pathological Characteristics of the Abdominal Aorta in AS Mice

As depicted in Figure 2b, the representative images show H & E-stained abdominal aortic tissue in longitudinal section from mice. In the control group, the intima of the abdominal aorta is smooth with no plaque formation, and the vessel wall thickness is uniform. In contrast, the vascular wall of mice in the model group exhibits significant protrusion into the lumen, resulting in distinct plaque and fibrous cap formation. However, in the experimental groups treated with *W. coagulans* JA845 and *W. coagulans* JA845 postbiotics, we observed that both were able to reduce the plaque formation rate in the abdominal aorta. Notably, the proportion of atherosclerotic plaques was significantly reduced after treatment with *W. coagulans* JA845 postbiotics, indicating a more potent effect of *W. coagulans* JA845 postbiotics in alleviating AS compared to *W. coagulans* JA845.

### 3.3. W. coagulans JA845 Postbiotics Regulates Expression of Immunological Markers in AS Mice

**Figure 3:**
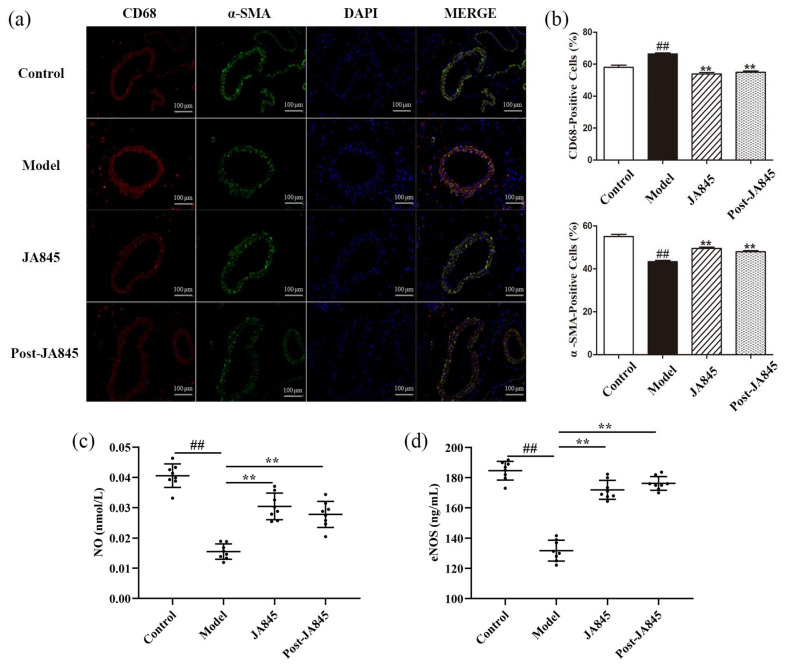
*W. coagulans* JA845 postbiotics improve the pathological characteristics of the abdominal aorta in AS mice (n = 8). (**a**) immunofluorescence, (**b**) CD68 and α-SMA-positive cell rate, and (**c**) NO reflect the influence of *W. coagulans* JA845 postbiotics on the expression of positive cell staining for CD68 and α-SMA immunological markers (n = 3). (**d**) eNOS reflects the effects of *W. coagulans* JA845 postbiotics on serum adhesion molecules. Results are manifested as the mean ± SD. Compared with the control group, ## *p* < 0.01, mean ± SD; compared with the model group, ** *p* < 0.01, mean ± SD.

Since foam cells primarily originate from smooth muscle cells (SMCs) and macrophages, the present study focuses on detecting the relative content of macrophages and SMCs in AS mice. We employed immunofluorescence staining techniques, targeting the α-smooth muscle actin (α-SMA) marker for SMCs and the CD68 marker for macrophages, to specifically label the abdominal aortic tissue of mice (Figure 3a). Through analysis of the staining results in Figure 3b, we observed that during the atherosclerotic process, the number of CD68-positive macrophages in the intimal region significantly increased, while the area of α-SMA-positive cells correspondingly decreased, both of which continuously promote disease progression. Following supplementation treatment with *W. coagulans* JA845 and *W. coagulans* JA845 postbiotics, the expression of CD68 decreased, while the expression of α-SMA increased, indicating that *W. coagulans* JA845 and *W. coagulans* JA845 postbiotics can mitigate abdominal aortic injury in AS mice.

### 3.4. W. coagulans JA845 Postbiotics Exerts an Impact on the Serum Adhesion Molecules Expression in AS Mice

To explore the suppressive impact of *W. coagulans* JA845 postbiotics on cell adhesion in atherosclerotic mice, we measured the concentrations of cell adhesion molecule within the aorta. The results are shown in Figure 3c,d, the levels of plasma NO and eNOS were significantly diminished in the model group compared to the control group mice. The intervention of *W. coagulans* JA845 postbiotics has demonstrated significant effects (*p* < 0.01).

### 3.5. W. coagulans JA845 Postbiotics Alleviate Inflammation in AS Mice

**Figure 4:**
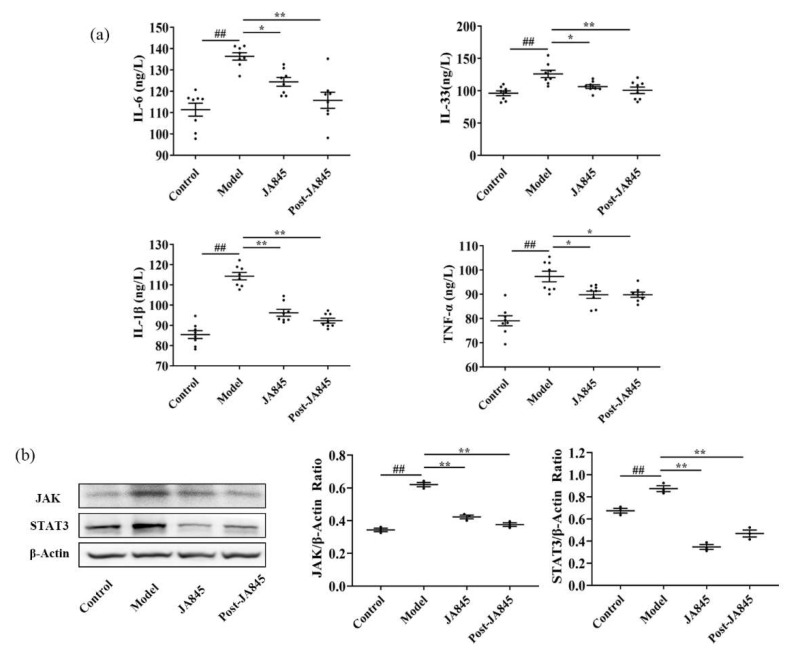
*W. coagulans* JA845 postbiotics alleviate inflammation in AS mice. (**a**) The levels of IL-6, IL-33, IL-1β, and TNF-α after A intervention (n = 8). (**b**) *W. coagulans* JA845 postbiotics inhibits activation and expression of JAK and STAT3 protein in AS mice and quantitative analysis. All the values are represented by the means ± SD. ## *p* < 0.01 versus control group; ** *p* < 0.01 and * *p* < 0.05 versus model group.

To investigate the regulatory mechanism of *W. coagulans* JA845 postbiotics on the inflammatory response in AS, the present study conducted a quantitative analysis of the levels of inflammatory factors in the serum of mice, specifically including IL-6, IL-33, IL-1β, and TNF-α (Figure 4a). The experimental results revealed that compared to the control group, the levels of inflammatory factors in the serum of AS model mice were significantly elevated (*p* < 0.01). However, after supplementation with *W. coagulans* JA845 and *W. coagulans* JA845 postbiotics, the levels of inflammatory factors exhibited a notable decrease. Notably, there were no statistically significant differences in the content of inflammatory factors between the JA845 and Post-JA845 groups (*p* > 0.05). This finding indicates that both *W. coagulans* JA845 and *W. coagulans* JA845 postbiotics can effectively regulate the levels of inflammatory factors in the serum of AS mice, thereby exerting a positive effect in reducing the inflammatory response.

### 3.6. W. coagulans JA845 Suppresses the Activation of the JAK/STAT3 Pathway in AS Mice

Given that the accumulation of trimethylamine *N*-oxide (TMAO), a metabolite of the gut microbiota, is significantly associated with the activation of protein expression in the JAK/STAT3 signaling pathway, and both together exhibit a trend of coordinated changes with inflammatory damage of endothelial cells, we conducted an in-depth analysis of protein expression levels in this signaling pathway. As depicted in Figure 4b, our research findings indicate that in the AS model, the combination of VD3, HFD, and choline water significantly aggravated the inflammatory response and activated the inflammasome pathway, specifically manifesting in a significant increase of 73.84 and 27.39% in JAK and STAT3 protein expression, respectively (*p* < 0.01). The supplementation with *W. coagulans* JA845 and *W. coagulans* JA845 postbiotics-intervention inhibited the progression of inflammation, leading to a significant reduction in JAK and STAT3 protein levels (*p* < 0.01).

### 3.7. W. coagulans JA845 Postbiotics Improves Gut Microbiota in AS Mice

**Figure 5:**
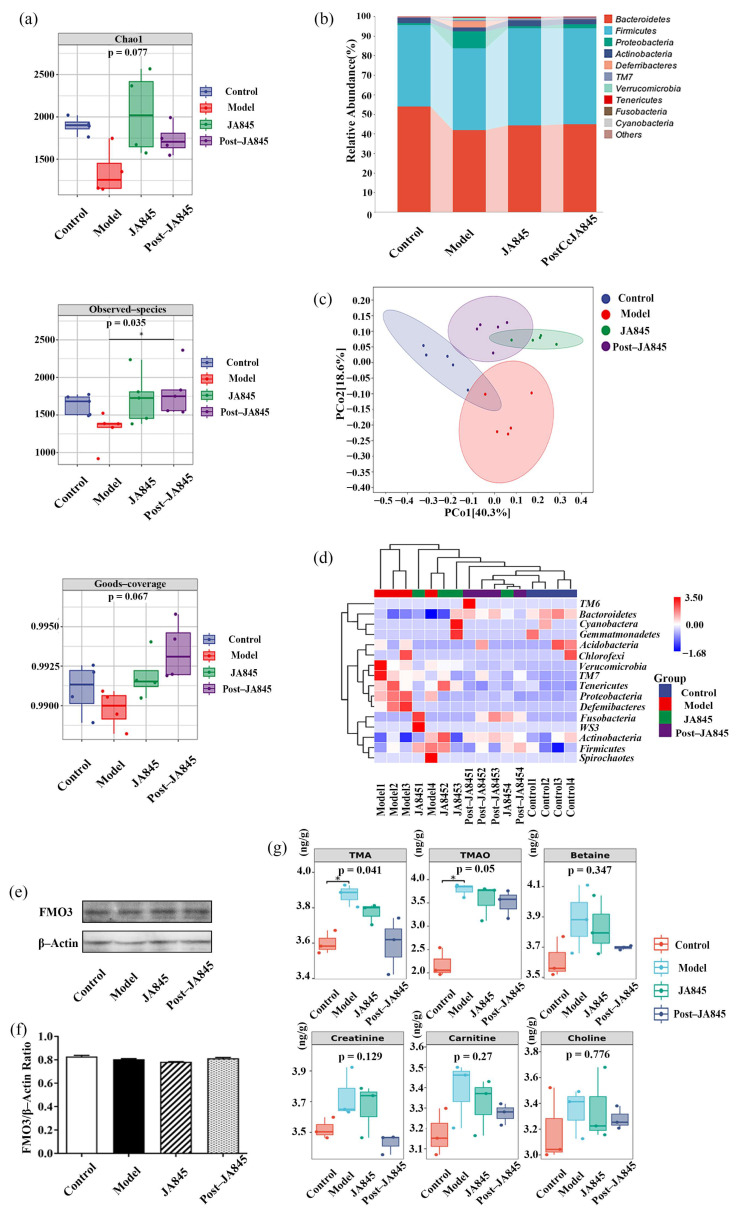
*W. coagulans* JA845 postbiotics-mediated changes of gut microbiota in HFD combined with VD3-induced AS mice (n = 5). (**a**) Alpha diversity plots, (**b**) species composition, (**c**) PCoA plot, and (**d**) heat map analysis of species composition. (**e**,**f**) Expression levels of related protein expression were determined by the Western blot, with quantitative analysis of the expression of FMO3. (**g**) Quantitative analysis of differential metabolites was carried out based on the LC-MS technique to investigate the effect of the postbiotics of Bacillus coagulans JA845 on the changes of metabolites. * *p* < 0.05.

According to alpha diversity analysis, the Chao1 observed-species (*p* < 0.05) and goods-coverage index of the gut microbiota of the mice in the *W. coagulans* JA845 and *W. coagulans* JA845 postbiotics groups were higher than those in the model group (Figure 5a). At the phylum level, the *W. coagulans* JA845 and *W. coagulans* JA845 postbiotics treatment increased the relative abundance of *Bacteroidetes* and *Firmicutes*. In addition, the relative abundance of *Firmicutes* in both of the treatment groups was lower than that in the control group (Figure 5b). Regarding β-diversity, the Principal Coordinates Analysis (PCoA) showed that the two axes, PCo1 and PCo2, accounted for 40.3% and 18.6% of the total variance, respectively. The gut microbial community composition of mice in the model group was clearly separated from that of the other two groups. This indicates that a high-fat diet, vitamin D3, and choline water led to significant changes in the microbial community, while the treatment with *W. coagulans* JA845 and the postbiotics of *W. coagulans* JA845 could partially reverse such changes (Figure 5c). Furthermore, this same group of samples exhibited distinct clustering patterns. Figure 5d illustrates the taxonomic phylum levels of the four groups examined in this study. The model group exhibited higher levels of *Verrucomicrobia*, *Proteobacteria,* and *Deferribacteria* but lower levels of *Bacteroidetes* compared to the control group. Notably, the supplementation of *W. coagulans* JA845 postbiotics resulted in a shift towards the microbiota compositions of the control group, which was characterized by an increase in the levels of *Bacteroidetes* and a decrease in the levels of *Verrucomicrobia*, *Proteobacteria,* and *Deferribacteria*.

### 3.8. W. coagulans JA845 Postbiotics Modulates Variation of Metabolites Between Groups in AS Mice

To evaluate the potential impact of *W. coagulans* JA845 postbiotics on TMAO synthesis in mice, we measured the concentrations of TMA, TMAO, Betaine, Creatinine, Carnitine, and Choline in cecal metabolites following *W. coagulans* JA845 postbiotics supplementation (Figure 5g). The results indicated that *W. coagulans* JA845 postbiotics supplementation led to a decrease in serum levels of TMA (*p* < 0.05), TMAO (*p* = 0.05), Betaine, Creatinine, Carnitine, and Choline. However, it is noteworthy that the therapeutic effect of *W. coagulans* JA845 was inferior to that of postbiotics. Simultaneously, the result showed that both did not affect the protein expression levels of FMO3 (Figure 5e,f).

### 3.9. W. coagulans JA845 Postbiotics-Mediated Combined Analysis of Intestinal Microbiota and Differential Metabolites in AS Mice

**Figure 6:**
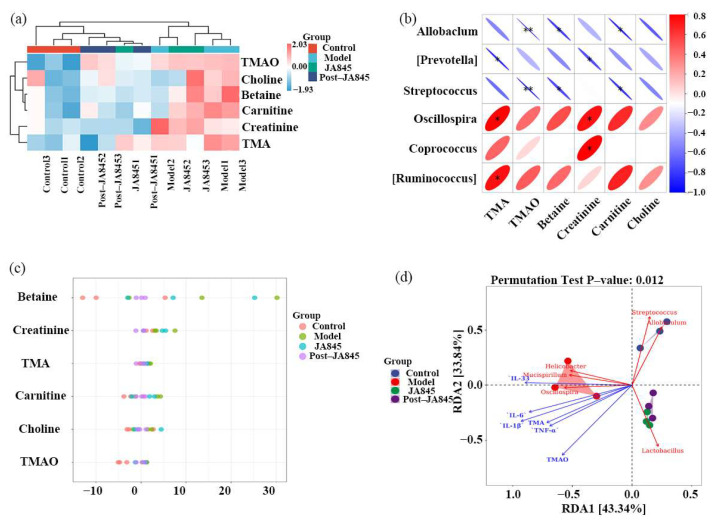
*W. coagulans* JA845 postbiotics-mediated combined analysis of intestinal microbiota and differential metabolites. (**a**) Species hierarchical cluster analysis. (**b**) Spearman correlation analysis. (**c**) Z-score plot. (**d**) Redundancy correlation analysis. ** *p* < 0.01 and * *p* < 0.05.

Species hierarchical cluster analysis (Figure 6a) revealed that the expression levels of TMA and TMAO in the model group were significantly higher than those in the control and treatment groups. After intervention with *W. coagulans* JA845 postbiotics, the expression levels were similar to those in the control group, and the control group clustered together with the *W. coagulans* JA845 postbiotics-intervention group. The Spearman correlation analysis was conducted to explore the relationship between the gut microbiota and key metabolites-TMA and TMAO (Figure 6b). The results indicate that *Akkermansia* and *Ruminococcus* are positively correlated with the levels of TMA and TMAO in feces, whereas *Allobaculum* and *Streptococcus* exhibit an opposite trend. The Z-score plot (Figure 6c) was used to measure and display the relative contents of metabolites across four groups, with consistent results and significant differences highlighted between the model group and the other three groups. To further investigate the relationship between genus-level differences in the gut microbiota of mice and the levels of TMA, TMAO, inflammatory markers (IL-6, IL-33, IL-1β, and TNF-α), and BAs, redundancy analysis (RDA) was performed (Figure 6d). The first two RDA axes, RDA1 and RDA2, explain 43.34% and 33.84% of the total variance, respectively, and significant differences were observed between the samples from different groups. Specifically, the *W. coagulans* JA845 and *W. coagulans* JA845 postbiotics groups showed a higher relative abundance of *Lactobacillus*, which was positively correlated with TMA and TMAO. In contrast, the control group had higher relative abundances of *Allobaculum* and *Streptococcus*, which were negatively correlated with TMA and TMAO. Additionally, the model group showed a higher relative abundance of *Helicobacter*, *Mucispirillum*, and *Oscillospira*, which were positively correlated with inflammatory factors.

## 4. Discussion

Postbiotics are bioactive substances that are derived from non-living bacterial products or metabolic byproducts of microorganisms, which exhibit various biological activities within the host organism [2,23]. By preparing *W. coagulans* postbiotics, the active components can be transported to the gut in a stable state and have a direct effect on it [24]. In the present study, we investigated the effects of the postbiotics from *W. coagulans* JA845 on gut microbiota dysbiosis induced by a high-fat diet combined with vitamin D3 and choline, which is associated with atherosclerosis [25].

Changes in the diversity and abundance of gut microbiota species are linked to an increased risk of atherosclerosis [26]. It has been observed that *Ruminococcus* is enriched in the model group, whereas *Bacteroides* and *Parabacteroides* are enriched in the normal group [27]. Studies have confirmed that *Bacteroides* can protect ApoE^−/−^ mice from atherosclerosis [28]. As expected, the addition of *W. coagulans* JA845 postbiotics increased the abundance of *Bacteroides* and *Parabacteroides* while reducing the abundance of *Ruminococcus*, thereby improving atherosclerosis. Similarly, *Lactobacillus paracasei* CCFM1224 postbiotics have been shown to suppress the relative abundance of *Ruminococcus* [29], effectively preventing the development of non-alcoholic fatty liver disease in mice. Consistent with these findings, the addition of *W. coagulans* JA845 postbiotics increased the abundance of *Bacteroides* and *Parabacteroides* while decreasing that of *Ruminococcus*, suggesting a positive impact of JA845 postbiotics on the gut microbiota composition in atherosclerotic mice. Moreover, the enrichment of *Akkermansia* has been shown to be beneficial for improving atherosclerosis [30]. However, in our study, the abundance of *Akkermansia* significantly decreased following treatment with JA845 postbiotics, which may be due to the competition between *Akkermansia* and other species that could inhibit the proliferation of *Akkermansia* [31]. Additionally, previous studies have reported that the levels of *Ruminococcaceae* in atherosclerosis patients and *Prevotella* in control groups are closely related to TMAO production [32].

Strategies to mitigate TMAO-induced cardiovascular risk primarily involve inhibiting FMO3 enzymatic activity to prevent TMA oxidation and modifying gut microbiota composition to reduce TMA generation at the source [33,34]. Studies have shown that probiotics reduce TMAO levels by modulating gut microbiota composition [35], independent of FMO3 expression, a finding consistent with our results. *W. coagulans* JA845 reduced TMA and TMAO levels in the cecum of AS mice without significant differences in FMO3 expression. This may be due to the fact that the regulatory effect of *W. coagulans* JA845 postbiotics on the gut microbiota plays a dominant role, rather than through direct inhibition of the activity of related enzymes, thereby achieving the effect of reducing trimethylamine *N*-oxide (TMAO). Similarly, Qiu et al. demonstrated that *Lactobacillus plantarum* reduced serum TMA and TMAO levels without affecting hepatic FMO3 expression [36]. Also, probiotics can lower serum TMA and TMAO concentrations by modulating gut microbiota structure [37], further reducing TMA levels in the gut and consequently decreasing TMAO levels [34], thereby helping to delay the onset of cardiovascular diseases. However, given the limited colonization ability of *W. coagulans* JA845 in the gut [38], we opted for postbiotic preparations as a strategy for treating atherosclerosis. Moreover, compared to its probiotic counterpart, JA845 postbiotics exhibited superior efficacy in reducing TMA and TMAO accumulation [5], likely due to their enhanced ability to restructure the gut microbial ecosystem. This effect may be attributed to the observed decrease in *Ruminococcus* abundance [39], as this genus has been positively correlated with TMAO production. By reshaping the gut microbiota environment [5,35], JA845 postbiotics may attenuate microbial enzymatic activity involved in TMA biosynthesis, thereby reducing systemic TMAO levels and mitigating its deleterious cardiovascular effects. These findings highlight the potential of postbiotics as a novel and effective intervention strategy for TMAO-associated cardiovascular pathologies.

TMAO exacerbates vascular inflammation by inducing foam cell and plaque formation [14]. Inflammatory responses play a central role in AS progression, with TMAO known to exacerbate inflammation via NLRP3 inflammasome activation and JAK/STAT3 pathway modulation [15,40]. Our study revealed that *W. coagulans* JA845 and its postbiotic treatments significantly reduced pro-inflammatory cytokines IL-6, IL-33, IL-1β, and TNF-α levels, exerting anti-inflammatory effects [41]. High IL-1β expression activates the JAK/STAT signaling pathway, amplifying the inflammatory cascade. The JAK/STAT3 pathway is a crucial regulator of endothelial dysfunction [42], vascular smooth muscle proliferation, and inflammatory cell infiltration. Also, *W. coagulans* JA845 postbiotics promoted eNOS production [43] and enhanced NO bioavailability [44,45], which are essential for vascular homeostasis and endothelial function maintenance. Our findings suggest that *W. coagulans* JA845 postbiotics alleviate AS-related inflammation via gut microbiota regulation and TMAO suppression, highlighting their therapeutic potential. It should be emphasized that research on this postbiotic remains in the preclinical phase as it has only been tested in animal models and not yet evaluated in human studies.

## 5. Conclusions

**Figure 7:**
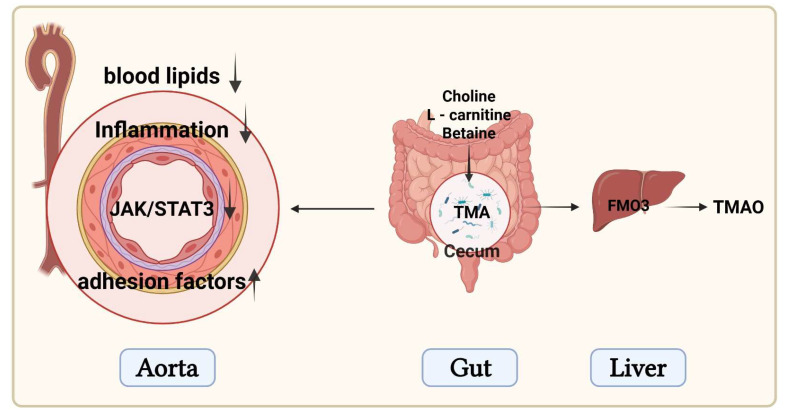
Schematic diagram of the mechanisms (Created in BioRender. Ma, L. (2025) https://BioRender.com/kyz5ayr).

In this study, we found that *W. coagulans* JA845 and its postbiotics exerted positive effects on atherosclerosis induced by a high-fat diet combined with vitamin D3 and choline water. Compared to *W. coagulans* JA845, the *W. coagulans* postbiotics significantly improved the composition of the gut microbiota, reduced the levels of TMAO, alleviated inflammation, protected vascular endothelial function, and enhanced the stability of atherosclerotic plaques. To sum up, the present study demonstrates that postbiotics mitigate atherosclerosis-related inflammation and improve vascular function through the gut-vascular axis by regulating TMAO (Figure 7).

## Data Availability

Data will be made available on request.
